# Knockdown of USF1 and USF2 drives prolonged changes in the gene expression response of M12-5B3 cells to DNA damage

**DOI:** 10.1371/journal.pone.0328438

**Published:** 2025-07-14

**Authors:** Kimberly S. Bellingham-Johnstun, Lisa A. Metzger, Jennifer L. Stone, Kevin Gerrish, Pierre R. Bushel, Michael L. Sikes

**Affiliations:** 1 Department of Biological Sciences, North Carolina State University, Raleigh, North Carolina, United States of America; 2 Molecular Genomics Core Laboratory, National Institute of Environmental Health Sciences, Research Triangle Park, North Carolina, United States of America; 3 Biostatistics and Computational Biology Branch, National Institute of Environmental Health Sciences, Research Triangle Park, North Carolina, United States of America; Zhejiang Cancer Hospital, CHINA

## Abstract

Therapeutic resistance remains a primary obstacle to curing cancer. Healthy cells exposed to genotoxic insult rapidly activate both p53-dependent and -independent non-genetic programs that pause the cell cycle and direct either DNA repair or apoptosis. Cancer cells exploit these same pathways as they respond to stresses induced by cancer therapies. In this study, we investigated a potential role for upstream stimulatory factor 1 (USF1) and USF2 in the p53-independent response of lymphoma cells to genotoxic therapy. We previously found that lymphocytes utilize the responsiveness of USF1 to double-stranded DNA breaks to coordinate T cell receptor beta (*Tcrb*) gene expression during V(D)J recombination. Here, microarray gene expression analysis of derivatives of the p53-deficient mouse B lymphoma cell line, M12, revealed that simultaneously depleting cells of both USF1 and USF2 altered the expression of 940 gene transcripts (>1.50-fold change, < 0.05 FDR), relative to cells expressing a scrambled control shRNA. Seven days after exposure to a single sublethal (5 Gy) dose of ionizing radiation, USF-depleted (USFKD) cells exhibited widespread and distinct transcriptional responses from those of irradiated controls (5035 and 5054 differentially expressed gene transcripts, respectively, with roughly half shared between both cell types). Gene ontology analyses revealed that USF knockdown induced numerous changes in the expression of genes critical for immune development and function while diminishing the responsiveness of genes linked to DNA damage pathways. Microarray findings were confirmed by RT-qPCR for a panel of genes responsive to USF knockdown and/or irradiation. These findings shed further light on transcriptional responses to ionizing radiation that manifest over time in transformed cells, identifying a novel p53-independent role in lymphocytic DNA damage stress responses for USF.

## Introduction

Cancer arises from genome instability that accumulates when improperly or incompletely repaired DNA lesions are replicated in cycling cells. To avoid genomic instability, cells activate a multi-faceted response to DNA damage that involves cell cycle arrest, DNA repair, metabolic regulation, and if necessary, permanent senescence or apoptosis. Though much of this activity is rapidly mobilized after DNA damage through actions coordinated by p53, recovery also includes a protracted p53-independent transcriptional response that is not well understood. Cancer cells exploit these same protective pathways to survive genotoxic chemotherapies, resulting in therapy resistant tumors that drive relapse and death [1]. Understanding the nature of DNA damage response (DDR) pathways, particularly those that function independent of p53, is critical to design of strategies to mitigate or overcome therapeutic resistance.

USF1 and USF2 were among the first mammalian transcription factors identified and have long been linked to cancer [2,3]. Their roles in pathology are complex. While loss of USF activity is implicated in multiple solid tissue cancers including breast [4], ovarian [5], prostate [6], oral epithelial [7], gastric, and skin cancers [8–10], multiple studies have paradoxically shown that USF1 in particular can promote cancers such as cutaneous squamous cell carcinoma, melanoma, cervical cancer, and osteosarcoma [11–15]. The Cancer Genome Atlas (TCGA) analyses of lung adenocarcinoma (LUAD) and liver hepatocellular carcinoma (LIHC) found that USF1 is highly expressed in patients with LUAD and LIHC and correlates with poor prognosis of LIHC patients [16,17]. USF proteins bind the promoters of other transcription factors and tumor suppressor genes [16,18–21], repress expression of TERT [22], and antagonize MYC [23–25]. Beyond its role as a transcription factor, USF1 binds and protects cytoplasmic p53 in skin cells from MDM2-dependent degradation when phosphorylated by UV-activated p38-MAPK [8,9,26]. *Helicobacter pylori* promotes gastric carcinogenesis in part through impairing nuclear localization of USF1:p53 complexes [10]. How such diverse responses sum to achieve USF’s regulatory role in cancer requires a more holistic map of the USF transcriptional circuits, but recent studies suggest that loss of USF provides proliferative and migratory advantages [27].

USF1 and USF2 are basic helix-loop-helix (bHLH) transcription factors that bind primarily as USF1/2 heterodimers at E boxes in the regulatory regions of over 2500 target genes [16,28]. Stress responsiveness of USF dimers is controlled via their phosphorylation by protein kinases including p38 and ERK MAPK, DNA-PK, GSK3β, CK2 and PKA [2]. Attempts to define the role of USF in stress responses and cancer development are complicated by an essential role for USF in differentiation, which results in embryonic lethality of mice that carry USF1/USF2 double knockout mutations or USF dominant negative transgenes, and by the ability of homodimers of either USF1 or USF2 to partially compensate for loss of the other’s activity in single gene knockout animals [29,30].

We previously found that DNA-PK-dependent phosphorylation of USF regulates germline transcription of *Tcrb* gene segments in response to dsDNA breaks (DSBs) generated either during V(D)J recombination or by exposure to sublethal doses of ionizing radiation [31]. Repeated induction of V(D)J recombinase in lymphocyte cultures resulted in USF activation that persisted weeks after the window in which nonhomologous end-joining (NHEJ) repair programs would be expected to resolve DNA lesions, and which was only restored to baseline by DNA-PK inhibition. Regulation of USF1 by DNA-PK was also shown in the liver of fasting mice where microbreaks generated during activation of fatty acid synthase transcription led to USF1 phosphorylation that was essential for *Fas* promoter activation [32]. Activation in response to DNA damage signals suggests a potential role for USF in adaptation to genotoxic stress.

In addition to DSB responses during V(D)J recombination, activated mature B lymphocytes must also faithfully process DSBs introduced during immunoglobulin gene somatic hypermutation (SHM) and class-switch recombination (CSR) as they optimize antibody structure and function [33]. Not surprisingly, these critical steps in B cell development are also primary drivers of non-Hodgkin lymphoma. To uncover USF’s contribution to the DNA damage response, we used RNA interference (RNAi) to simultaneously deplete both USF1 and USF2 from M12-5B3, a recombinase-inducible derivative of the p53-deficient B lymphoma cell line M12 [34,35]. M12-5B3 is fixed at the germinal center stage of B cell development, circumventing the lethality of depleting both USFs in developing cells, and also lacks p53 which might mask more subtle USF-dependent responses. M12-5B3 cells were transduced with either pScramble, a scrambled shRNA control lentivirus or a mixture of USF1 and USF2 shRNA viruses (hereafter labeled USFKD). Stably transduced clonal lines were established based on their resistance to lentiviral-encoded puromycin and exposed to a single 5 Gy dose of ionizing radiation (IR). The transcriptomic response to USF depletion and ionizing radiation was measured using Affymetrix GeneChip transcriptome arrays.

Prior to irradiation, microarray analysis identified several hundred differentially expressed genes (DEGs), primarily associated with lymphoid function, in USF-depleted cells. IR appeared to accentuate transcriptional changes induced by USF knockdown alone when assessed one day after exposure but drove more widespread and divergent gene expression patterns at seven days post-IR. Even at this later time, the majority of DEGs in USFKD cells were associated with immune functions. In contrast, DEGs associated with DNA repair, chromatin organization and apoptosis induced seven days after IR were largely limited to control cells and absent or diminished in USF knockdowns. Microarray findings were confirmed by RT-qPCR for a number of immune-related genes not previously identified as putative USF targets including *Il2rg, Blk, Blnk, Foxj1, Cdkn1b, Aicda* and *Nfkbid,* among others. The impact of depleting either USF1 or USF2 alone varied with regard to the target gene and was less impactful than depleting both gene products, in agreement with partial functional overlap between USF1 and USF2 [30]. These findings demonstrate that USF proteins play a significant role in lymphocyte transcriptional regulation and that loss of USF limits the p53-independent response to DNA damage days after insult.

## Methods

### Cell culture

The murine M12-5B3 RAG-inducible germinal center B cell line and its culture has been described previously [35,36]. Its identity was verified for this study by expression analyses (RT-qPCR) of the IgH, Igκ and Igλ genes, and by sequence analysis of its previously defined Igκ Vκba9*/Jκ5 rearrangement [37]. For this study, cells were maintained in the presence of tetracycline (0.5 μg/ml) to prevent expression of tet-inducible RAG1 and GFP-RAG2 transgenes. RAG silencing was confirmed in all subsequent experiments by fluorescence microscopy, absence of RT-qPCR products for RAG transgenes, and the absence of PCR products for Igλ gene assembly [38]. For treatment with ionizing radiation, cells were seeded at a density of 3 x 10^5^/ml culture medium in 10 cm dishes the day before treatment. The next day, cells were harvested into 15 ml conical centrifuge tubes and exposed to a single sublethal dose (5 Gy) of ionizing radiation using a Gammacell 220 cobalt-60 irradiator (MDS Nordion). After irradiation, cells were resuspended in fresh culture medium and allowed to recover for the specified number of days.

### RNA interference and transfection

For depletion of USF2 in M12-5B3 cells, a validated Mission USF2 shRNA (TRCN0000071575) in pLKO.1 was purchased from Sigma. Two independent USF1 targeting shRNAs were designed using the BROAD Institute Genetic Perturbation Platform. Duplexed shRNA USF1shR1 and USF1shR2 oligonucleotides (S1 Table) were cloned into the AseI site of pLKO.1, a gift from David Root (Addgene plasmid #10878) [39], and insertion was verified by sequence analysis (data not shown). The sshRNA plasmid in which a scrambled shRNA sequence was cloned into the AseI site of pLKO.1 was a gift from David Sabatini (Addgene plasmid #1864) [40].

For shRNA plasmid transfections, 1 x 10^7^ 293T cells were seeded per 10 ml DMEM complete medium (supplemented with 10% fetal calf serum, 2 mM L-glutamine, 0.01% penicillin/streptomycin, and 50 mM β-mercaptoethanol) and allowed to attach for 4 hours. pUSF1shR1, pUSF1shR2 and pUSF2shR (640 ng each) or psshRNA (2.56 μg) was mixed together with pFG12 (640 ng), pMDLG (1.6 μg), pREV (1.6 μg) and pCMV-G (1.0 μg) in 870 μl serum free DMEM and 56 μl FugeneHD (Roche), vortexed briefly, and allowed to complex at room temperature for 10 minutes before dropwise addition to plated cells. The next day, culture medium was replaced with complete DMEM supplemented with 1% BSA. Culture supernatant was harvested at 24 and 36 hours later and filtered at 0.45μm.

For lentiviral transductions, viral supernatant (500 μl) supplemented with polybrene (8 μg/ml final concentration) was added to 10^5^ M12-5B3 cells seeded in 500 μl complete RPMI 1640 (10% fetal calf serum, 2 mM L-glutamine, 0.01% penicillin/streptomycin, and 50 mM β-mercaptoethanol), and the plated cells were centrifuged (1 hr @ 1200 x g @ RT). GFP reporter fluorescence was confirmed in transduced wells one day after transduction, and cells were selected with the addition of puromycin (3 μg/ml final concentration).

### Western blotting

To prepare nuclear protein isolates, cells were lysed in NP-40 Buffer (500 μl of 10 mM HEPES pH 7.9, 1.5 mM MgCl_2_, 10 mM KCl, 0.5 mM DTT, 0.1% NP-40) supplemented with PMSF and HALT protease and phosphatase inhibitor cocktail (Thermo Scientific) for 2 min on ice. After lysis, cells were pelleted in a microcentrifuge (2 min @ 12,000 RPM @ 4^o^C), the nuclear pellet was resuspended in nuclear lysis buffer (100 μl 10 mM HEPES pH 7.9, 25% glycerol, 0.42 M NaCl, 1.5 mM MgCl2, 0.2 mM EDTA, 0.5 mM DTT) supplemented with PMSF and HALT protease and phosphatase inhibitor cocktail, and incubated 30 min on ice with manual agitation every 2 min. Nuclear lysate was recovered by microcentrifugation (20 min @ 12,000 RPM @ 4^o^C) and stored at -80^o^C.

Nuclear lysates (15 μg) were subjected to sodium dodecyl sulfate (SDS)-polyacrylamide gel electrophoresis (PAGE) and Western blot analysis. ECL Prime (GE Healthcare) was used to detect specific protein signals per the manufacturer’s instructions, followed by autoradiography. Rabbit anti-mouse polyclonal antibodies (Santa Cruz Biotechnology) against USF1 (sc-229), USF2 (sc-862) and Sp1 (sc-59) and goat anti-rabbit HRP secondary antibody (Pierce, 32260) were used as recommended by the provider. Fold knockdown was calculated using ImageJ analysis of autoradiography films as previously described [41,42].

### Affymetrix microarray hybridization

Total cellular RNA was extracted from 1−5 x 10^6^ cells using RNeasy Plus (Qiagen) according to the manufacturer’s protocol. RNA levels were quantified using a NanoDrop 1000 spectrophotometer (Thermo Scientific) and stored at -80^o^C. After confirming RNA quality on an AATI Fragment Analyzer (Advanced Analytical Technologies, Ankeny, IA), gene expression analysis in each of three independent replicates per treatment condition was conducted using Affymetrix Mouse Transcriptome 1.0 arrays (Affymetrix, Santa Clara, CA). Total RNA (100 ng) for each sample was amplified and labeled as directed using the Affymetrix WT Plus Reagent Kit (WT Plus Kit). Amplified biotin-cDNAs (5.5 μg) were fragmented and hybridized to each array for 16 hours at 45°C in a rotating hybridization oven. Array slides were stained with streptavidin/phycoerythrin utilizing a double-antibody staining procedure and then washed for antibody amplification according to the GeneChip Hybridization, Wash and Stain Kit and user manual following protocol FS450−0001. Arrays were scanned in an Affymetrix Scanner 3000 and data was obtained using the GeneChip® Command Console software. The microarray data is available on NCBI’s GEO database (Geo accession: GSE101846).

### Bioinformatic analysis of gene expression

CEL files were preprocessed by computing the robust multiarray average (RMA) of background adjusted, quantile normalized and log2 transformed perfect match (PM) pixel intensity values [43,44]. Microarray analysis was performed using a three-way (cell line, treatment, and time) analysis of variance (ANOVA) model with a three-way interaction term in Partek Genomics Suite v6.6 software. For each cell line, DEG transcripts were detected by contrasting treatment group means versus the mean of the untreated controls or sshRNA 1d IR and using a FDR < 0.05 with an absolute fold change >1.50. The DEGs were enriched for biological pathways using gene ontology (GO) biological processes (BP) and the Kyoto Encyclopedia of Genes and Genomes (KEGG) knowledge base [45] in the Database for Annotation, Visualization, and Integrated Discovery (DAVID) v6.7 Bioinformatics resource [46,47]. To visually represent the 152 GO biological processes enriched by the statistically significant DEG transcripts in each of the indicated contrasts, the –log10 p-value of each BP term was computed using the functional enrichment Fisher Exact Test. Missing values were imputed as 0 (−log base 10(1), i.e., p-value = 1). Euclidean distance as the dissimilarity metric and average linkage grouping were used for clustering the BPs in the rows.

### TopGO motif discovery

To identify biological processes affected by USF knockdown we used topGO v2.58.0 [48] and the mta10transcriptcluster.db v8.8.0 microarray gene annotation database to enrich the 1,078 gene symbols obtained from the USFKD IR 7days vs WT IR 7days contrast DEG transcripts. We used the Fisher statistic with the weight algorithm in topGO to account for the topology and hierarchy of the Gene Ontology. Finally, we used the Hypergeometric Optimization of Motif EnRichment (HOMER) software v5.1 [49] with default settings (e.g., −300 to +50 relative to the transcription start site, motif lengths 8, 10, 12) to identify motifs in the promoters of the DEGs obtained from the USFKD 7d IR vs sshRNA no IR contrast. The promoters of the genes were based on *Mus musculus,* mm9 assembly RefSeq genes sequence from −2000 to +2,000 relative to the transcript start site (TSS). Selection criteria for known motifs were based on the following filters: % background with motif > 3% and < 10%, % target with motif > 5%, enrichment > 1.3, and q-value < 0.01. The top motifs found *de novo* were based on the least probable false positives and the selection criteria used for the known motifs except for q-value.

### Quantitative PCR

For RT-qPCR analyses, each RNA sample (1 μg) was reverse transcribed using RevertAid MMLV reverse transcriptase (Thermo Scientific) and oligo d(T) primers. The resultant cDNAs were amplified in triplicate qPCR reactions with 1 μmol each of the indicated primer pairs (S1 Table). Triplicate qPCR reactions (20 μl SensiMix Plus; Bioline) were amplified (94^o^C, 20 sec; 57^o^C, 30 sec; 72^o^C, 30 sec) for 40 cycles, followed by melt-curve analysis using a MyiQ2 iCycler (Bio-Rad). Target gene expression was calculated by ΔΔ threshold cycle (C_T_) normalization to untreated sshRNA controls and standardized for loading variations by comparison with values obtained for β-actin.

## Results

### USF depletion

**Fig 1:**
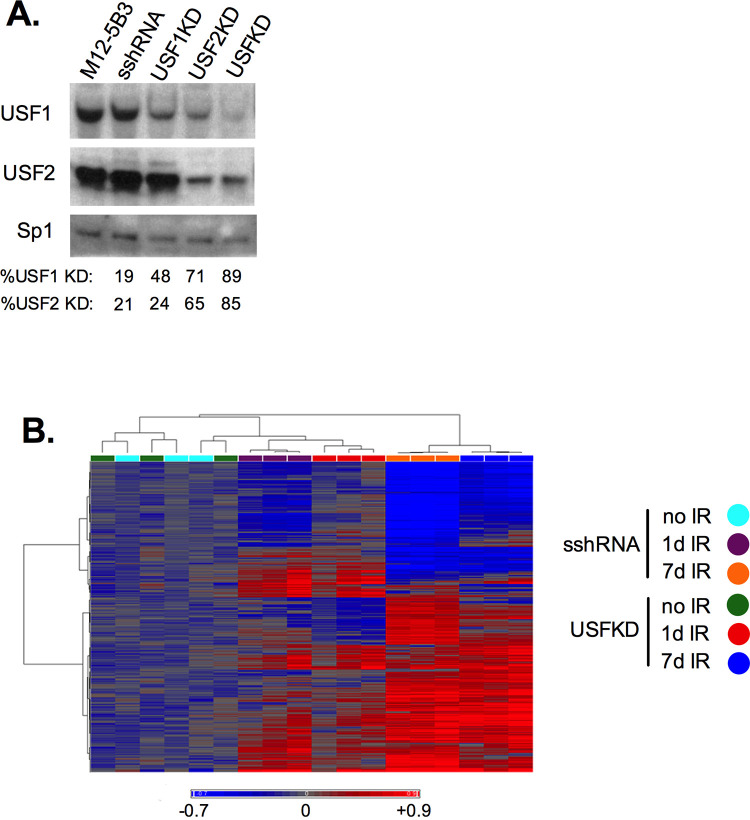
Transcriptomic response to USF depletion and ionizing radiation. (A) USF knockdown in M12-5B3 cells. Western blot analysis of USF1 (upper panel) and USF2 nuclear protein levels (middle panel) in stable USF-depleted cells relative to parental M12-5B3 and sshRNA-transduced control cells. Sp1 protein levels (lower panel) control for variations in protein loading. Fold knockdown levels of USF1 and USF2 are shown below. (B) Heatmap of differentially expressed gene (DEG) transcripts detected by Affymetrix GeneChip whole transcriptome arrays in USF-depleted (USFKD) and control (sshRNA) clones (n = 3) before treatment (no IR), or one day (1d IR) and seven days after ionizing radiation (7d IR). Data represents the log2 ratio of RMA-normalized intensity data for each sample relative to the mean of the genotype-matched no IR samples. Red color represents increased expression, blue represents decreased expression and grey denotes no change. Spearman rank as the similarity measure and Ward grouping were used for clustering of gene transcripts in the rows (left) and samples in the columns (top).

To investigate a potential role for USF in the p53-independent transcriptional response to DNA damage, we used lentiviral-mediated RNAi to simultaneously deplete USF1 and USF2 proteins from the mouse B lymphoma cell line, M12-5B3. Puromycin-resistant clones expressing short-hairpinned RNAs targeting either *Usf1* or *Usf2* alone or in combination, or those transduced with a scrambled shRNA (sshRNA) control were isolated and screened for nuclear USF protein levels by Western blotting (Fig 1A). Previous studies in USF2^-/-^ mice showed that loss of USF2 led to reduced USF1 expression [20]. Consistent with this finding, we observed 2 to 3.5-fold knockdown of USF1 protein levels in cells transduced with shRNAs targeting either *Usf1* or *Usf2* (USF1KD or USF2KD), but 9-fold USF1 reduction in cells transduced with shRNAs targeting both *Usf* RNAs (USFKD). Depletion of USF2 protein expression was only observed in the presence of *Usf2* shRNAs, ranging from 3 to 7-fold in the USF2KD and USFKD clones, respectively. No overt phenotypic changes in sshRNA or USF knockdown cultures were observed (i.e., their relative proliferation and turnover rates were similar to those of parental M12-5B3, data not shown).

### Transcriptomic analysis of USF depletion

To map USF’s role in the transcriptional response to DNA damage, we exposed USFKD and sshRNA cultures (n = 3) to a single sublethal (5 Gy) dose of ionizing radiation. Perhaps owing to the p53-deficiency of M12, both sshRNA and USFKD cultures showed minimal toxicity to the 5 Gy exposure, with initial arrest by one day and return to growth by 6–7 days. No overt differences between the control and knockdown cells were apparent during the treatment window (data not shown). Total RNA from untreated cells and cells allowed to recover from IR treatment for either one day or seven days was profiled on Affymetrix Mouse Transcriptome 1.0 arrays (Fig 1B). Using this approach, we identified 9289 DEG transcripts showing> or < 1.50-fold change (<0.05 FDR) between samples. To account for both USF knockdown and treatment conditions, DEG transcripts were modeled using a 3-way ANOVA (cell line, treatment and time) with a three-way interaction term (cell line*treatment*time) first to partition the variance (between groups and within groups). Next, data from the samples were contrasted by groups for comparison. Less than 10% of the total DEG transcripts identified in the study were present in untreated USFKD cells, corresponding to 235 transcripts with increased expression and 705 transcripts with decreased expression in USFKD 7d IR relative to USFKD no IR when compared with untreated sshRNA (S2A and B Fig).

During the first day of recovery, the majority of transcriptional responses to IR are p53-dependent [50]. Absent p53, sshRNA only exhibited 360 DEG transcripts that met the + /-1.50-fold change and <0.05 FDR thresholds of significance in pairwise contrasts between no IR samples and 1d IR samples. Radiation induced more extensive differences at one day in the USFKD cells, which showed 2268 statistically significant DEG transcripts relative to untreated sshRNA. However, none of these DEG transcripts rose to the level of significance when the USFKD 1d IR samples were contrasted with USFKD no IR cells at the < 0.05 FDR threshold, suggesting that irradiation primarily accentuated preexisting differences caused by USF removal. USF-dependent transcriptional responses were much more evident seven days post-IR. When normalized to sshRNA no IR, 7d IR sshRNA and USFKD each showed markedly different patterns of gene expression at seven days (Fig 1B). Pairwise contrasts between sshRNA no IR and either sshRNA 7d IR or USFKD 7d IR revealed 5054 or 5035 significant DEG transcripts, respectively. Moreover, unlike the contrast between USFKD no IR and 1d IR, contrast between USFKD no IR and USFKD 7d IR revealed 2615 significant DEG transcripts unique to the 7d IR samples. Of 2962 genes upregulated in sshRNA 7d IR and 3184 upregulated in USFKD 7d IR relative to sshRNA no IR, only 1421 genes were shared between the two (S2C Fig). Likewise, of the 2092 genes downregulated in sshRNA 7d IR cells and 1851 genes downregulated in USFKD 7d IR, only 1063 were shared between the two cell lines (S2D Fig). The scope of USF’s involvement in transcriptional responses seven days after IR is further underscored when the USFKD 7d IR expression pattern is compared with that of USFKD no IR: 93% and 64% of the upregulated and downregulated DEG transcripts respectively in USFKD are only observed in the 7d IR cells.

### Gene Ontology clustering analysis

**Fig 2:**
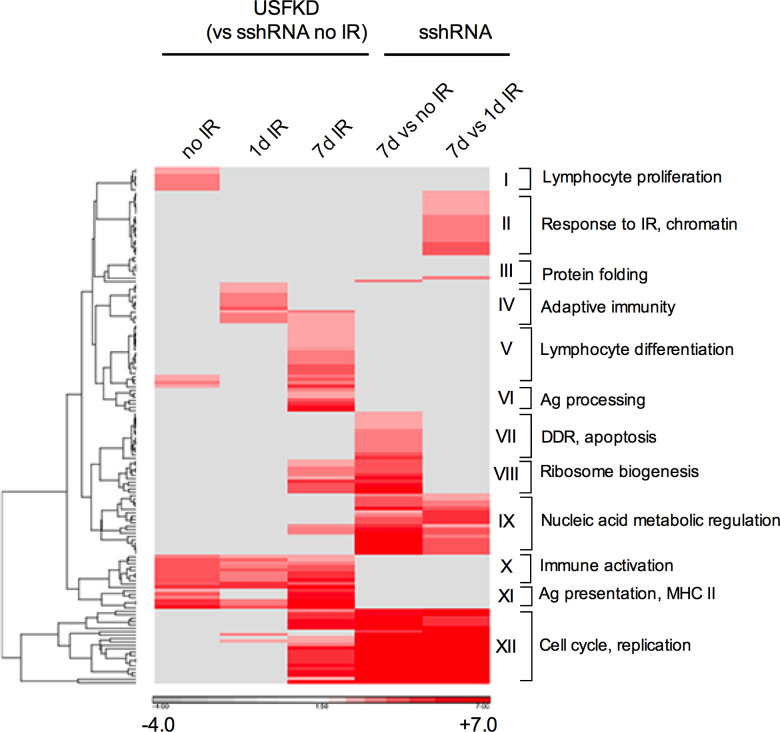
Heat map of the clustering among the enriched Gene Ontology biological processes terms annotated to the DEGs. Heatmap representation of the clustering of 152 Gene Ontology (GO) biological process (BP) terms enriched (FDR < 0.05) by the statistically significant DEG transcripts in each of the indicated contrasts. The data represents the –log10 p-value of each BP term from the functional enrichment Fisher Exact Test. Missing values were imputed as 0 (−log base 10(1), i.e., p-value = 1). Euclidean distance as the dissimilarity metric and average linkage grouping were used for clustering the BPs in rows. Twelve enrichment clusters are each labeled with the BP that best represents the biological theme associated with the enriched GO terms. Darker red coloring signifies greater enrichment, while grey represents no enrichment.

We next used GO and KEGG knowledge bases to move beyond single gene analyses and identify biological pathways enriched with DEGs in each experimental condition. Clustering analyses identified 152 distinct GO terms associated with DEGs in the study, which we grouped into 12 clusters of related biological function (Fig 2). A complete list of GO terms and DEGs associated with each cluster is provided in S2 Table. DEGs resulting from USF depletion in M12-5B3 largely clustered with immunity-related biological processes. Irrespective of treatment, DEGs associated with immune functions (clusters IV, V, X, and XI) including MHC II-dependent antigen processing and presentation (cluster XI), antigen receptor production (cluster IV), and lymphocyte differentiation (cluster V), were enriched in USF-depleted cells. DEGs associated with proliferation of immune cells and protein phosphorylation (cluster I) were only enriched in untreated USFKD cells, while DEGs associated with the assembly of large multiprotein or macromolecular complexes (cluster VI) were only enriched in USFKD samples seven days after IR treatment. TopGO allowed us to look at the biological processes associated with DEGs in the USFKD 7d IR samples relative to the sshRNA 7d IR samples (Table 1). The top 20 annotated GO terms were each very strongly associated with immune-specific biological functions such as antigen processing and presentation, cytokine production, kinase activity, and lymphocyte proliferations, activation and maturation. Similar results were obtained using KEGG pathway enrichment analysis but are more focused on disease-specific pathways (S3 Table). To gain an initial sense of the potentiality for USF knockdown directly or indirectly affecting expression of the identified DEGs, we screened the promoter regions (−300 bp to +50 bp) of DEGs in the USFKD 7d IR vs sshRNA no IR contrast for stringent binding motifs of 8, 10, and 12 nucleotides using Hypergeometric Optimization of Motif EnRichment (HOMER) (S4 Table). USF1 and USF2 motifs were significantly enriched in DEG promoter regions relative to background, as were the motifs for other bHLH transcription factors. Binding motifs for ETS family members ETS1, PU.1, and SpiB were also enriched. There is no evidence suggesting USF regulates expression of these factors. In contrast, each is extensively involved in many of the biological functions identified in Table 1.

**Table 1:** TopGO annotation of biological processes affected by USF knockdown.

GO ID	GO Term	Genes Annotated	Genes Significant	Expected	P-value
GO:0002478	Antigen processing and presentation of exogenous peptide antigen	33	13	0.98	4.6e-12
GO:0002503	Peptide antigen assembly with MHC class II protein complex	11	8	0.33	9.4e-11
GO:0050670	Regulation of lymphocyte proliferation	264	31	7.81	1.9e-09
GO:1902679	Negative regulation of RNA biosynthetic process	1317	78	38.98	7.0e-09
GO:0031343	Positive regulation of cell killing	106	17	3.14	1.7e-08
GO:0002709	Regulation of T cell mediated immunity	120	18	3.55	5.7e-08
GO:0030183	B cell differentiation	157	19	4.65	5.3e-07
GO:0009617	Response to bacterium	964	58	28.53	8.8e-07
GO:0002428	Antigen processing and presentation of peptide antigen via MHC	37	9	1.09	1.1e-06
GO:0050864	Regulation of B cell activation	147	20	4.35	1.5e-06
GO:0032609	Type II interferon production	144	17	4.26	1.5e-06
GO:0002467	Germinal center formation	16	6	0.47	4.4e-06
GO:0002486	Antigen processing and presentation of endogenous peptide antigen via MHC class I via ER pathway, TAP-independent	35	8	1.04	7.1e-06
GO:0002767	Immune response-inhibiting cell surface receptor signaling pathway	6	4	0.18	1.1e-05
GO:0071901	Negative regulation of protein serine/threonine kinase activity	90	12	2.66	1.5e-05
GO:0007040	Lysosome organization	107	13	3.17	1.9e-05
GO:0002923	Regulation of humoral immune response mediated by circulating immunoglobulin	20	6	0.59	1.9e-05
GO:0032653	Regulation of interleukin-10 production	65	10	1.92	2.2e-05
GO:0043405	Regulation of MAP kinase activity	159	16	4.71	2.4e-05
GO:0001819	Positive regulation of cytokine production	543	38	16.07	3.1e-05

Whereas USF depletion altered the expression levels of immune-related genes, it also impaired the differential expression of genes associated with DDR seven days into IR recovery. GO associated with response to ionizing radiation and DNA damage (II and VII), DSB repair (II), chromatin regulation (II), and apoptosis (VII) were enriched in sshRNA 7d IR but were not detected in USFKD 7d IR cells (Fig 2). Both sshRNA and USFKD cells were enriched seven days after IR for GO terms associated with mRNA and rRNA processing, intracellular transport and ribosome biogenesis (VIII and IX), as well as multiple terms associated with cell cycle progression, mitosis and DNA replication (XII). Taken together, these findings suggest that USF activity is required in M12-5B3 to regulate many genes involved in multi-day responses to genotoxic stress.

USF proteins direct the repression or activation of numerous tissue-specific genes. Indeed, USF1 regulation of genes associated with antigen presentation and recognition was also observed in *Usf1*^*-/-*^ mice [51]. To investigate the broad impact of USF depletion on lymphocyte-specific functions, we surveyed the DEGs enriched in our GO analyses. Of the 25 genes that showed the largest increase (Table 2) or decrease in expression after USF

**Table 2:** Top 25 annotated genes upregulated in USFKD.

RefSeq	Gene Symbol	Annotation	Fold-Change	p-Value
NM_153558	Obp2a	Odorant binding protein 2A	6.07	1.79E-11
NM_178036	Lcn10	Lipocalin 10	4.41	4.09E-10
NM_009645	Aicda	Activation-induced cytidine deaminase	3.47	3.83E-11
NM_010130	Emr1	EGF-like module-containing mucin-like hormone receptor-like 1	3.09	1.00E-11
NM_008976	Ptpn14	Protein tyrosine phosphatase non-receptor type 14	2.73	2.89E-11
NM_031843	Dpp7	Dipeptidylpeptidase 7	2.65	1.73E-09
NM_170758	Cd300a	CD300A antigen	2.61	2.03E-08
NM_001166835	Vmn1r79	Vomeronasal 1 receptor 79	2.60	1.72E-05
NM_009627	Adm	Adrenomedullin	2.52	3.27E-07
NM_178440	Myo1g	Myosin IG	2.49	1.72E-08
NM_007549	Blk	B lymphoid kinase	2.48	1.25E-06
NM_001167539	Vmn1r238	Vomeronasal 1 receptor 238	2.47	3.97E-05
NM_001167534	Vmn1r2	Vomeronasal 1 receptor 2	2.39	2.04E-05
NM_009626	Adh7	Alcohol dehydrogenase 7 (class IV) mu or sigma polypeptide	2.36	1.15E-07
NM_008528	Blnk	B cell linker	2.36	1.96E-07
NM_009906	Tpp1	Tripeptidyl peptidase I	2.28	2.62E-09
NM_172142	Nfkbid	Nuclear factor of kappa light polypeptide gene enhancer in B cells inhibitor delta	2.26	6.71E-09
NM_001111026	Runx1t1	Runt-related transcription factor 1; translocated to, 1 (cyclin D-related)	2.26	3.62E-08
NM_001136062	Eno3	Enolase 3, beta muscle	2.25	7.57E-10
NM_172600	Tmem260	Transmembrane protein 260	2.23	4.82E-09
NM_008634	Map1b	Microtubule-associated protein 1B	2.19	1.60E-07
NM_001164563	Amigo2	Adhesion molecule with Ig like domain 2	2.15	6.28E-08
NM_153175	Gimap6	GTPase, IMAP family member 6	2.15	2.58E-08
NM_001033308	Themis2	Thymocyte selection associated family member 2	2.10	1.79E-07
NM_178911	Pld4	Phospholipase D family, member 4	2.09	2.01E-09

Genes differentially up-regulated in untreated USFKD cells over sshRNA controls.

knockdown (Table 3), multiple play essential roles in lymphocyte development, maintenance, and function. Activation induced cytidine deaminase (AID) directs SHM and CSR activities in germinal center B cells. The *Aicda* gene was overexpressed in USFKD cells, as were *Dpp7* (which encodes a proline dipeptidase that facilitates apoptotic resistance of resting lymphocytes), *Myo1g* (which encodes a hematopoietic cell-specific myosin), *Cd300a* (which encodes the CD300A inhibitory immune receptor), and the genes for B lymphoid kinase (*Blk*) and B cell linker (*Blnk*) that mediate Ig signaling, suggesting a role for USF in limiting their

**Table 3:** Top 25 annotated genes downregulated in USFKD.

RefSeq	Gene Symbol	Annotation	Fold-Change	p-Value
NM_013563	Il2rg	Interleukin 2 receptor, gamma chain	−2.85	1.03E-07
NM_001166397	Armcx2	Armadillo repeat containing, X-linked 2	−2.86	2.60E-13
NM_001081079	Ogfrl1	Opioid growth factor receptor-like 1	−2.92	1.54E-10
NM_009514	Vpreb3	Pre-B lymphocyte gene 3	−2.96	1.18E-08
NM_001081084	Cubn	Cubilin (intrinsic factor-cobalamin receptor)	−2.99	1.40E-10
NM_001168693	Endou	Endonuclease, polyU-specific	−3.09	9.26E-13
NM_183264	Tespa1	Thymocyte expressed, positive selection associated 1	−3.11	1.43E-10
ENSMUST00000082422	mt-Tt	Mitochondrially encoded tRNA threonine	−3.16	3.00E-04
NM_001177767	Rex2	Reduced expression 2	−3.19	1.85E-09
NM_145562	Parm1	Prostate androgen-regulated mucin-like protein 1	−3.25	1.74E-11
NM_001114383	Luzp4	Leucine zipper protein 4	−3.26	2.01E-08
NM_016972	Slc7a8	Solute carrier family 7 (cationic amino acid transporter, y + sys	−3.68	1.60E-11
NM_007807	Cybb	Cytochrome b-245, beta polypeptide	−3.68	4.88E-09
ENSMUST00000082400	mt-Tc	Mitochondrially encoded tRNA cysteine	−3.77	1.43E-05
NM_153171	Rgs13	Regulator of G-protein signaling 13	−4.00	3.95E-09
NM_183390	Klhl6	Kelch-like 6	−4.12	1.47E-11
NM_001163085	Map3k15	Mitogen-activated protein kinase kinase kinase 15	−4.18	6.19E-11
NM_019791	Maged1	Melanoma antigen, family D, 1	−4.20	2.58E-12
NM_146234	Mmgt1	Membrane magnesium transporter 1	−4.22	8.76E-12
NM_007979	F9	Coagulation factor IX	−4.34	2.46E-12
NM_172785	Zc3h12d	Zinc finger CCCH type containing 12D	−4.58	7.29E-12
ENSMUST00000082401	mt-Ty	Mitochondrially encoded tRNA tyrosine	−5.07	2.32E-05
NM_011845	Mid2	Midline 2	−5.08	7.80E-12
NM_007799	Ctse	Cathepsin E	−6.05	1.43E-11
NM_012009	Sh2d1b1	SH2 domain protein 1B1	−8.04	1.11E-13

Genes differentially down-regulated in untreated USFKD cells over sshRNA controls.

expression. Conversely, USF knockdown reduced expression of genes encoding interleukin 2 common gamma chain (*Il2rg*), kelch-like 6 (*Klhl6*), regulator of G-protein signaling 13 (*Rgs13*) and Transformed Follicular Lymphoma protein (*Zc3h12d*).

### RT-qPCR validation of transcriptomic data

**Fig 3:**
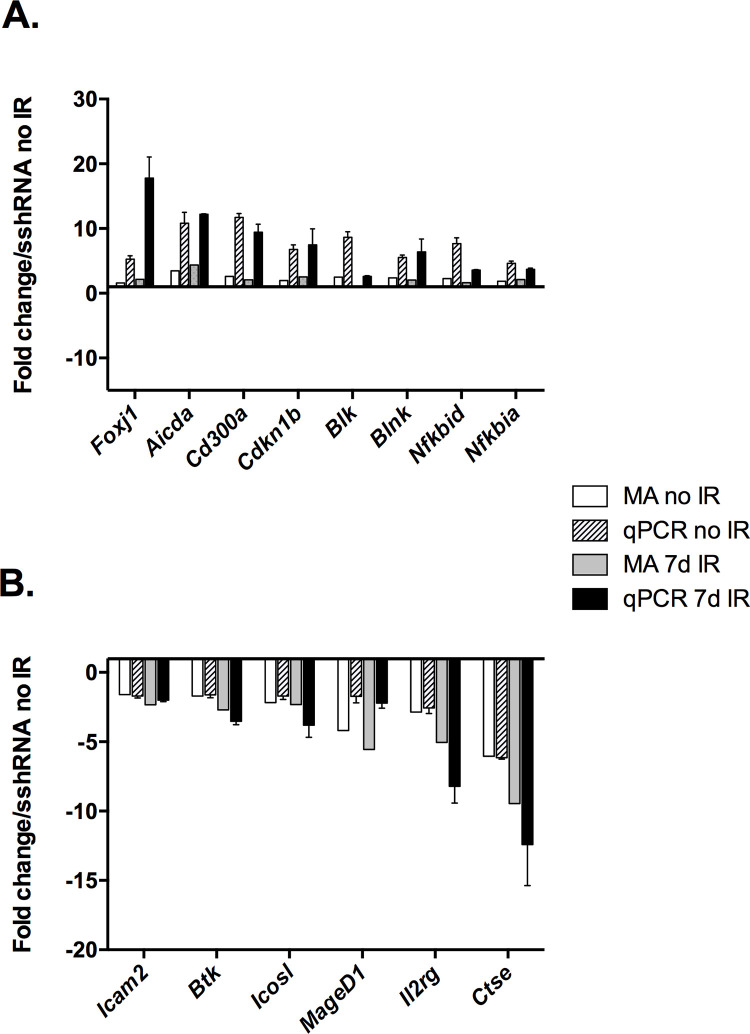
Quantitative PCR (qPCR) validation of microarray findings. A panel of genes indicated by microarray (MA) as differentially expressed in USFKD relative to sshRNA were analyzed by RT-qPCR. Bars for microarray data represent average fold change in the indicated samples relative to sshRNA no IR. Bars for RT-qPCR represent average fold increase (A) or decrease (B) in expression (±SD, n = 3) of the indicated genes in USFKD no IR or USFKD 7dIR relative to sshRNA no IR. Relative RT-qPCR signals were calculated by ΔΔC_T_ and then normalized to β-actin loading controls. Values shown are representative of two independent experiments.

We used RT-qPCR to measure expression levels in separate biological replicates of untreated and seven days post-IR sshRNA and USFKD cells, targeting a panel of genes that were identified as differentially expressed by microarray and associated with B cell activation and/or cancer. In each case, we were able to recapitulate the directionality of altered gene expression in USFKD cells relative to sshRNA no IR controls (Fig 3), while increased sensitivity of the qPCR revealed a greater magnitude of differential expression between USF-depleted and control cells than was evident in the microarray. For example, while microarray showed that the immunomodulatory *Foxj1* transcription factor gene was expressed in USFKD no IR and USFKD 7d IR cells at 1.58- and 2.17-fold above sshRNA no IR control, respectively, qPCR indicated increased expression levels of 5.28 ± 0.51 and 17.80 ± 3.24, (Fig 3A). qPCR revealed similar increases in the expression of *Aicda* (10.81 ± 1.69 and 12.21 ± 0.08), *Cd300a* (11.71 ± 0.62 and 9.41 ± 1.25)*, Blnk,* (5.54 ± 0.37 and 6.40 ± 1.98) and *Nfkbid* (7.68 ± 0.90 and 3.56 ± 0.10), which encodes IκBNS. qPCR showed decreases in the expression of *Il2rg* (−2.56 ± 0.40 and −8.21 ± 1.21) and the Cathepsin E gene *Ctse* (−6.14 ± 0.11 and −12.41 ± 2.97).

**Fig 4:**
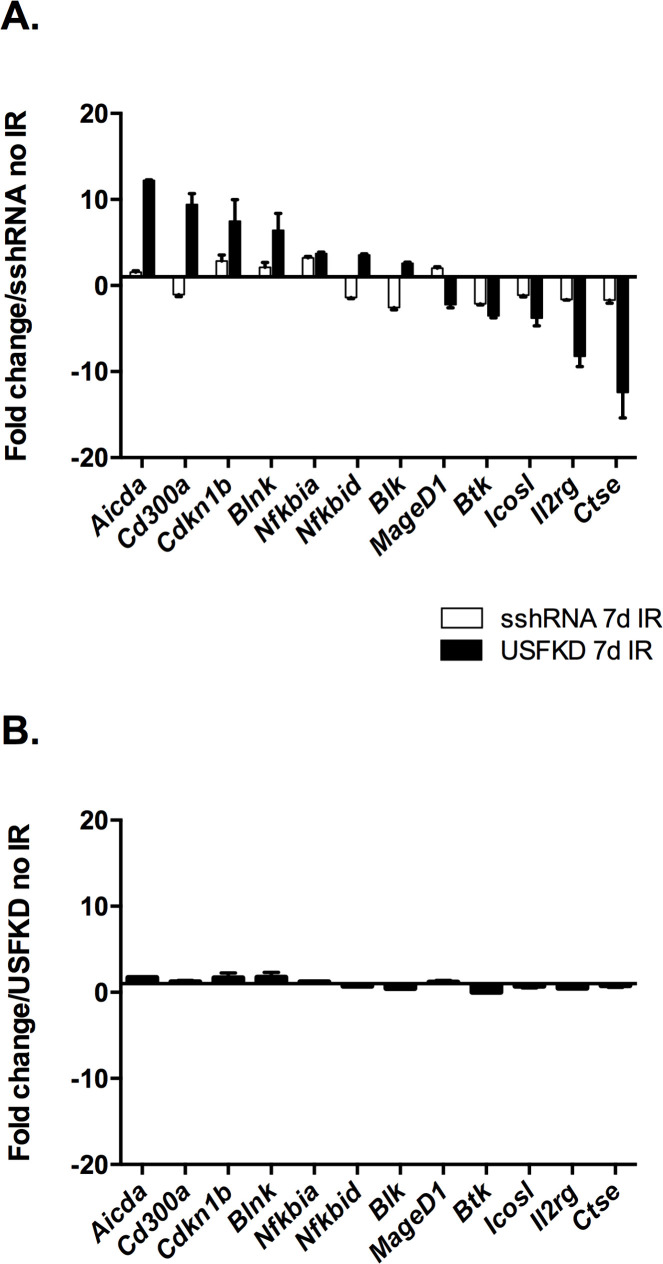
USF knockdown alters expression of immune-related genes. RT-qPCR was used to compare the expression of select DEGs in USFKD and sshRNA 7d IR cultures that were also altered in USFKD no IR. Bars represent average fold change in expression (±SD, n = 3) of the indicated genes in sshRNA 7d IR (white) and USFKD 7d IR cells (black) relative to levels in sshRNA no IR (A) or USFKD no IR (B). qPCR signals for each 7d IR sample were calculated by ΔΔC_T_ relative to the appropriate no IR control, normalized to β-actin controls and are representative of two independent experiments.

The genes screened by qPCR were selected based on their relevance to humoral immunity and their altered expression upon USF depletion prior to irradiation. When we used qPCR to compare expression levels in the control and knockdown cells seven days after IR (Fig 4), we found that differential expression of *Aicda, Cd300a, Cdkn1b, Blnk, Nfkbid, Blk* and *Icosl* was largely restricted to USFKD when both sshRNA and USFKD 7d IR samples were normalized to sshRNA no IR. Quantitative PCR showed increased expression of *Aicda* and *Cd300a*, and reduced expression of *Il2rg* and *Ctse* exclusively in USFKD 7d IR, relative to sshRNA 7d IR. To determine if irradiation further impacted immune-related gene expression beyond USF depletion, we next normalized USFKD 7d IR levels to USFKD no IR (Fig 4B). Irradiation had little impact on expression of the selected genes, suggesting that USF depletion was primarily responsible for their differential expression.

**Fig 5:**
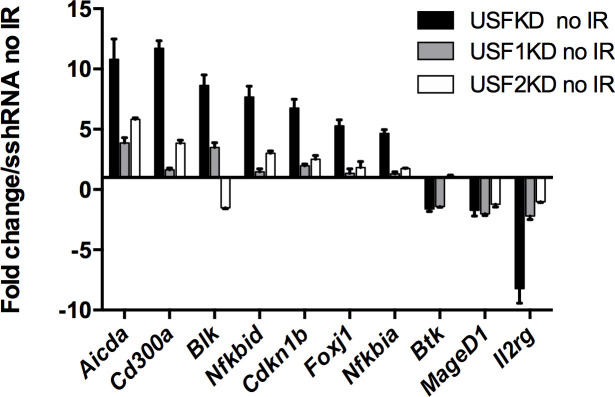
USF *trans-*activating function is only partially impaired when only one USF protein is depleted. RT-qPCR was used to contrast the impact on gene expression of simultaneously targeting USF1 and USF2 (black bars), with targeting either USF1 (USF1KD, grey bars) or USF2 (USF2KD, white bars) alone. Bars represent average fold change in expression (±SD, n = 3) of the indicated genes in untreated USFKD, USF1KD and USF2KD cells relative to levels in untreated sshRNA. Relative qPCR signals were calculated by ΔΔC_T_ and normalized to β-actin controls and are representative of two independent experiments.

Although USF binds DNA primarily as a heteromeric assembly of USF1 and USF2, homomers of either USF1 or USF2 can at least partially compensate for loss of the other protein [29]. Given that most genes identified as differentially expressed in USFKD cells have not previously been identified as either direct or indirect USF target genes, we compared gene expression in untreated USFKD cells with that in untreated cells singly depleted of either USF1 or USF2 (Fig 5). With the exceptions of *Btk* and *Maged1*, depleting both USF1 and USF2 resulted in a greater impact on target gene expression than depleting either USF alone. Expression of *Aicda, Cd300a* and *Nfkbid* genes was more strongly upregulated after USF2 depletion than after USF1 depletion, perhaps due to reduced expression of USF1 in USF2KD cells (Fig 1A). Conversely, expression of *Blk* and *Il2rg* was more significantly affected by depleting USF1. Differences in the expression of *Cdkn1b, Foxj1 and Nfkbia* between USF2KD and USF1KD cells were negligible. Together, these data agree with previous findings that USF transcriptional activity is generally mediated by heteromeric assemblies of USF1 and USF2, though each USF can partially compensate for loss of the other [28,30].

## Discussion

In this study we have demonstrated that USF regulates prolonged p53-independent transcriptional responses to DNA damage. USF1 and USF2 were among the first mammalian transcription factors identified. USF activity is modulated at multiple levels by a series of stress responsive kinases, and USF dysregulation, both gain and loss, has been linked to numerous cancer types [2,11–17]. We previously showed that DNA-PK signaling of USF1 can persist in lymphocyte cultures long after DNA damaging events [31]. To map the scope of USF involvement in the DNA damage response we characterized the transcriptional profile of M12-5B3 cells depleted of USF1 and USF2 before, one day after, and one week after their exposure to sublethal ionizing radiation. M12 cells carry a loss of function point mutation in the DNA binding domain of p53 [34], allowing us to focus on p53-independent transcriptional responses to IR. Although knockdown of the USF proteins alone was sufficient to alter expression of several hundred genes in M12-5B3, the scope of differential expression expanded roughly ten-fold when cells were stressed by irradiation and allowed to recover for one week. Very few of these DEGs have been previously reported as USF targets, though ChIP-seq studies have suggested USF binds over 2400 human genes [16,28]. The profile of observed DEGs further suggests that USF regulates a number of lymphocyte-specific transcriptional programs, but also that loss of USF dysregulates the transcriptional responsiveness to genotoxic stress.

Lymphocytes must pass through distinct developmental stages that involve the production and resolution of double stranded DNA breaks in their antigen receptor genes. Lymphocyte tolerance for such double-stranded breaks (DSBs) depends upon several factors including the tightly regulated expression of enzymes that drive V(D)J recombination, somatic hypermutation, and class-switch recombination, the antigen receptor gene-delimited scope of DSB production, and the efficiency of DNA repair mechanisms. At the same time, these transient physiological DNA breaks, signaling through ATM and DNA-PK regulate the expression of genes that counteract aspects of DNA damage response (DDR)-like pro-apoptotic responses, as well as many genes independent of the canonical DDR but essential for lymphocyte development [52,53]. Our findings here, together with our previous results demonstrate that USF proteins contribute to the lymphocyte DNA damage response through their regulation of diverse gene targets that include both DNA repair pathways and lymphocyte development and activation pathways.

USF1 and USF2 have independently been linked to the DDR. In addition to DSB-dependent phosphorylation of USF1 by DNA-PK, UVB radiation signals p38-MAPK phosphorylation of USF1 [26,31,32]. Indeed, loss of USF1 or its p38-mediated phosphorylation impairs the UVB DNA damage response in skin cells [8]. USF2 was independently identified as a transcriptional regulator of the DDR. Irradiation of USF2-deficient cells showed RNA-seq profiles consistent with weakened DDR and increased senescence [54]. Consistent with roles for both USFs in the DDR, we find that loss of either USF was sufficient to alter expression of multiple genes including *Nfkbia* and *Cdkn1b*. Likewise, expression of genes critical to lymphocyte development or function like *Aicda, Blk, Btk,* and *Il2rg* was altered in both USF1KD and USF2KD cells, though double knockdowns consistently showed a larger change in gene expression than either single knockdown. The impact of USF depletion on genes like *Aicda, Cdkn1b,* and *Il2rg* was present even before cells were irradiated, suggesting that USF may normally limit the responsiveness of some genes. In contrast, the restriction of other DEGs to sshRNA 7d IR suggests that for those genes USF is required for IR responsiveness.

Our findings illustrate two aspects of the USF-dependent DNA damage response: persistence of its impact on the genome days after DNA damage, and the scope of its involvement in regulating lymphocyte-specific gene expression. USF is pleiotropic, functioning as a chromatin insulator, a recruitment hub for other transcriptional elements, and a classical bHLH transcription factor to activate or repress a wide array of genes including multiple transcription factors [16,22,31,32,51,55]. Given this diverse functionality and regulation of other transcription factors, the broad scope of DEGs seen in USFKD cells seven days into IR recovery is not surprising. The ability of USF to function as both a chromatin insulator and a hub for recruitment of other transcriptional elements to a particular promoter might suggest that the persistent muting of DSB transcriptional responses in USFKD cells seven days after IR reflects a stable nonresponsive epigenetic structure in the absence of USF at those genes. However, our previous analysis of USF1-mediated repression control at the *Tcrb* gene suggests an alternative explanation. In that study, repeated induction of V(D)J recombinase in thymocyte cultures led to loss of USF1 binding at the 5’Dβ2 promoter that persisted for weeks to months in culture and was only restored by inhibition of DNA-PK [31]. Persistent stressors like unresolved DSBs impinge on protein phosphorylation/dephosphorylation cascades, dysregulating cell signaling particularly in transformed cells [56]. Indeed, sustained loss of USF function has been reported in multiple tumor types [4–8,10]. Studies of human ovarian cancer and murine *Helicobacter pylori* driven gastric cancer both showed cytoplasmic localization of USF1 [5,10]. In PC-3 human prostate cancer cells, HDAC inhibition blocked the nuclear accumulation of USF1 induced by ionizing radiation, leading to downregulation of USF1 target genes [57]. Further studies are needed to fully understand the influence of persistent stress on USF function and its contribution to pathologies such as the development and treatment resistance of cancer.

USF depletion from M12-5B3 led to significant changes in the expression of genes like *Klhl6, Aicda, Dpp7* and *Cd300a* that have been associated with B cell function and oncogenic pathology, but not associated with USF regulation [58–61]. Proline dipeptidase 2, the product of *Dpp7*, contributes to survival of resting B cells, while KLHL6 and CD300a are both involved in B cell activation and AID drives immunoglobulin somatic hypermutation and class-switch recombination programs in the germinal center after B cell activation. Given the diversity of DEGs identified in this study, we expect that USF depletion in other tissues would similarly enrich for DEGs specific to that tissue. Regardless, our findings are consistent with multiple studies have identified USF regulation of immune genes, particularly those associated with immune cell development and function [16,31,62–66].

## Conclusion

In conclusion, our findings support a role for USF in the prolonged transcriptional response of B lymphoma cells to DNA damage. In cells depleted of both USF1 and USF2, genes associated with B cell development and function, including *Aicda, Cd300a, Blk* and *Il2rg*, are dysregulated even in the absence of DNA damage. However, one week after exposure to IR, USF-depleted cells show expanded dysregulation of immune-related genes, while the transcriptional response of genes associated with DNA repair, chromatin regulation and apoptosis is impaired, suggesting that prolonged removal of USF activity may compromise aspects of the protective DNA damage response. Future studies will be necessary to unravel the complex interplay between the USFs, their regulatory kinases, and their target genes, and to define their contributions to lymphocyte activity and pathology.

## Supporting information

S1 Fig Raw images. Uncropped and unprocessed films of western blots for USF1 (A) and Sp1 and USF2 (B). To create the final figure for Fig 1A, the images were adjusted to grayscale, lane 1 (molecular weight markers) and lane 6 (unrelated mouse T cell line) were cropped out, relevant protein band region was selected, and contrast was minimally adjusted for clarity.
(TIF)

S2 Fig Gene expression response to USF depletion and IR. (A and B) Venn diagrams of DEG transcripts that exhibited >1.50-fold increase (A) or >1.50-fold decrease (B) in expression in either USFKD no IR or USFKD 7d IR cells relative to sshRNA no IR. Numbers of DEG transcripts present only in untreated samples (green circles), 7d IR samples (salmon circles) or in both (overlap region) are indicated (C and D) Venn diagrams of genes that exhibited >1.50-fold increase (C) or >1.50-fold decrease (D) in expression in USFKD 7d IR and sshRNA 7d IR relative to sshRNA no IR controls. Numbers of DEG transcripts present only in sshRNA 7d IR samples (green circles), USFKD 7d IR samples (salmon circles) or in both 7d IR samples (overlap region) are indicated.
(TIF)

S1 Table Oligonucleotides used in this study. (DOCX)

S2 Table GO terms and DEGs associated with twelve biological processes clusters. (XLSX)

S3 Table KEGG pathway enrichment analysis. (XLSX)

S4 Table HOMER DNA motif enrichment analysis. (XLS)

## Abbreviations

USF: Upstream stimulatory factor
RNAi: RNA interference
IR: ionizing radiation
shRNA: short-hairpinned RNA
sshRNA: scrambled short-hairpinned RNA M12-5B3 control cell line
USFKD: USF1/USF2 double knockdown M12-5B3 cell line
FDR: false discovery rate
DEG: differentially expressed gene.
