# Supplementary figures and images for: Knockdown of USF1 and USF2 drives prolonged changes in the gene expression response of M12-5B3 cells to DNA damage
Source: PLoS One. 2025 Jul 14;20(7):e0328438. doi: 10.1371/journal.pone.0328438 (PMC12258588; doi:10.1371/journal.pone.0328438)

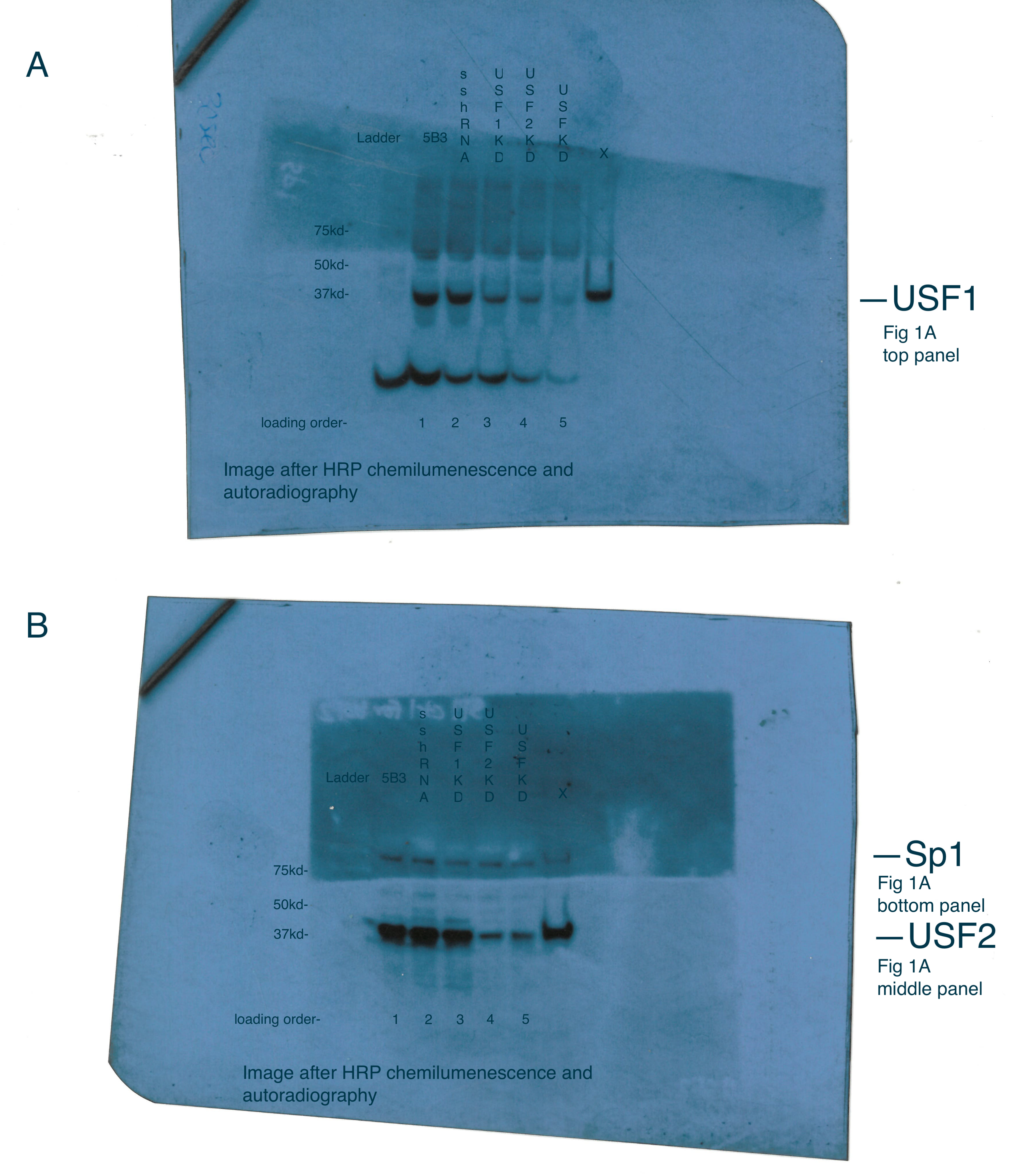

Supplement: S1 Fig — Uncropped and unprocessed films of western blots for USF1 (A) and Sp1 and USF2 (B). To create the final figure for Fig 1A, the images were adjusted to grayscale, lane 1 (molecular weight markers) and lane 6 (unrelated mouse T cell line) were cropped out, relevant protein band region was selected, and contrast was minimally adjusted for clarity. (TIF) [file pone.0328438.s001.tif]

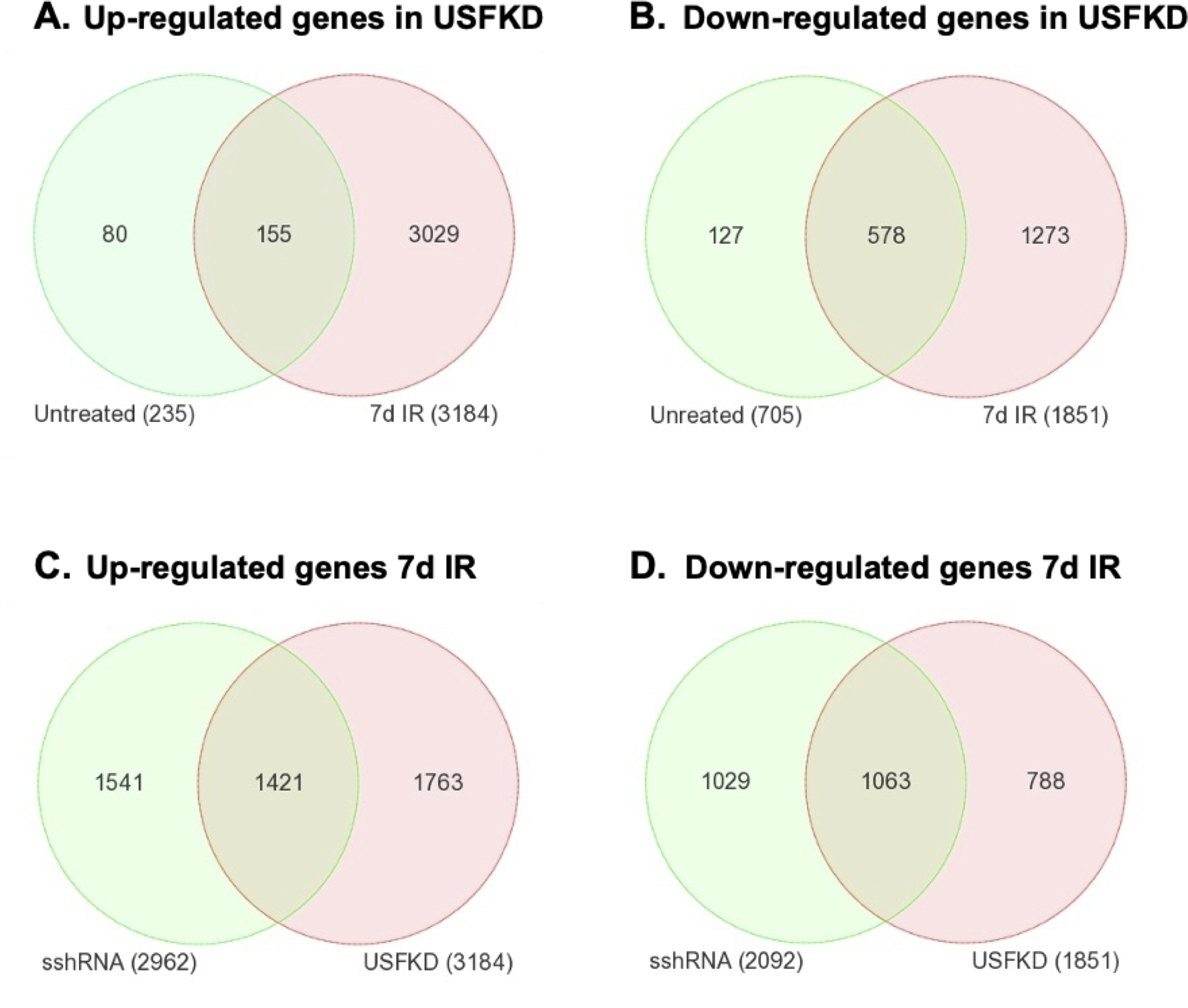

Supplement: S2 Fig — (A and B) Venn diagrams of DEG transcripts that exhibited >1.50-fold increase (A) or >1.50-fold decrease (B) in expression in either USFKD no IR or USFKD 7d IR cells relative to sshRNA no IR. Numbers of DEG transcripts present only in untreated samples (green circles), 7d IR samples (salmon circles) or in both (overlap region) are indicated (C and D) Venn diagrams of genes that exhibited >1.50-fold increase (C) or >1.50-fold decrease (D) in expression in USFKD 7d IR and sshRNA 7d IR relative to sshRNA no IR controls. Numbers of DEG transcripts present only in sshRNA 7d IR samples (green circles), USFKD 7d IR samples (salmon circles) or in both 7d IR samples (overlap region) are indicated. (TIF) [file pone.0328438.s002.tif]
