# Supplementary material for: Knockdown of USF1 and USF2 drives prolonged changes in the gene expression response of M12-5B3 cells to DNA damage
Source: PLoS One. 2025 Jul 14;20(7):e0328438. doi: 10.1371/journal.pone.0328438 (PMC12258588; doi:10.1371/journal.pone.0328438)
Supplement: S1 Table — (DOCX) [file pone.0328438.s003.docx]

| **S1 Table: Oligonucleotides used in this study.** | | |
| --- | --- | --- |
| **Primer** | **Sequence 5' to 3'** | |
| *USF1-targeting shRNAs* | | |
| USF1shR1 F | CCGGCAGAAGTTAAGATGCGTACCTCTCGAGAGGTACGCATCTTAACTTCTGTTTTTG | |
| USF1shR1 R | AATTCAAAAACAGAAGTTAAGATGCGTACCTCTCGAGAGGTACGCATCTTAACTTCTG | |
| USF1shR2 F | CCGGGAGGGCTCAACATAACGAAGTCTCGAGACTTCGTTATGTTGAGCCCTCTTTTTG | |
| USF1shR2 R | AATTCAAAAAGAGGGCTCAACATAACGAAGTCTCGAGACTTCGTTATGTTGAGCCCTC | |
| *qPCR Primers* | | |
| Aicda F | | ΑCACCTCCTGCTCACTGGACT |
| Aicda R | | GGTCCAGGTCCCAGTCTGA |
| Cdkn1b F | | TTGGACCAAATGCCTGACTC |
| Cdkn1b R | | GGGAACCGTCTGAAACATTTTC |
| Nfkbid F | | TCTTTCCCATTCTCTGCTTCTG |
| Nfkbid R | | AGGGAAGGCTCAGGATACAG |
| Blk F | | TCTGTTTGACTATGCCGCTG |
| Blk R | | CATAACCTTCTCTTCCTGTGACG |
| Cd300a F | | CAGGACCAACACTAGAGACAC |
| Cd300a R | | CAGGAGAGCTAACACAGACAA |
| Blnk F | | GAGGATGAGGCTGATTATGTGG |
| Blnk R | | GTGCTTTGAGGAACTGTTTGG |
| Ctse F | | TCGCAGTCCGACACATACAC |
| Ctse R | | CATCCACAGTCAACCCTTCC |
| Icosi F | | AGCCACAGAGTTAGTCAAGATC |
| Icosi R | | CATGCAGGTGTAGGTACGTTC |
| Nfkbia F | | AGGAGTACGAGCAAATGGTG |
| Nfkbia R | | CGGCTTCTCTTCGTGGATG |
| Foxj1 F | | CCATCTACAAGTGGATCACGG |
| Foxj1 R | | TGTTCAAGGACAGGTTGTGG |
| Icam2 F | | TGGAGAACAGGAATGGAAGC |
| Icam2 R | | TCGGTTGTGGAGATTGGTG |
| Irak1 F | | AGACTTTGCTGGCTACTGTG |
| Irak1 R | | AAGAATGTCCAGTCGTTGAGG |
| Btk F | | TGGAGAACAGGAATGGAAGC |
| Btk R | | TCGGTTGTGGAGATTGGTG |
| MageD1 F | | AGGAAACAGCGAAGGGATTG |
| MageD1 R | | CTGTCTTCAACAGAGGCCT |
| Il2rg F | | ACGGTACACATTTCGGGTTC |
| Il2rg R | | CAGCTTCCAGTGCAAACAAG |
